# Comparative Cytogenetics of the Malagasy Ground Geckos of the *Paroedura bastardi* and *Paroedura picta* Species Groups

**DOI:** 10.3390/ani14111708

**Published:** 2024-06-06

**Authors:** Marcello Mezzasalma, Gaetano Odierna, Rachele Macirella, Elvira Brunelli

**Affiliations:** 1 Department of Biology, Ecology and Earth Science, University of Calabria, Via P. Bucci 4/B, 87036 Rende, Italy; rachele.macirella@unical.it (R.M.); elvira.brunelli@unical.it (E.B.); 2 Independent Researcher, Via Michelangelo 123, 81031 Aversa, Italy

**Keywords:** evolution, karyotype, sex chromosomes, Madagascar, reptiles

## Abstract

**Simple Summary:**

Chromosome changes represent important events in evolution. They may trigger processes of speciation or be the result of phylogenetic diversification. In both cases they can represent discrete evolutionary markers of taxonomic significance. In this contribution, we performed a comparative cytogenetic analysis on several representatives of the Malagasy ground geckos of the genus *Paroedura*. Our results show that chromosome variability in this genus involves chromosome number, morphology, and the independent differentiation of sex chromosome systems in distinct evolutionary lineages. We also highlight that the taxonomic, genetic and chromosome diversity in *Paroedura* is still underestimated.

**Abstract:**

We present a comparative chromosome study of several taxa of the Malagasy ground geckos of the *Paroedura bastardi* and *P. picta* species groups. We employed a preliminary molecular analysis using a trait of the mitochondrial 16S rRNA gene (of about 570 bp) to assess the taxonomic status of the samples studied and a cytogenetic analysis with standard karyotyping (5% Giemsa solution), silver staining (Ag–NOR staining) and sequential C-banding (C-banding + Giemsa and + fluorochromes). Our results show that all the taxa studied of the *P. bastardi* group (*P. ibityensis*, *P. rennerae* and *P.* cf. *guibeae*) have a similar karyotype composed of 2n = 34 chromosomes, with two metacentric pairs (1 and 3) and all other pairs being acrocentric. Chromosome diversification in the *P. bastardi* group was mainly linked to the diversification of heteromorphic sex chromosome systems (ZZ/ZW) in *P. ibityensis* and *P. rennerae*, while no heteromorphic sex chromosome pair was found in *P.* cf. *guibeae*. The two taxa investigated of the *P. picta* species group (here named *P. picta* and *P.* cf. *picta* based on molecular data) showed the same chromosome number of 2n = 36, mostly acrocentric elements, but differed in the number of metacentric elements, probably as a result of an inversion at chromosome pair 2. We highlight that the genus *Paroedura* is characterized by the independent diversification of heterogametic sex chromosomes in different evolutionary lineages and, similarly to other phylogenetically related gecko genera, by a progressive formation of a biarmed element by means of tandem fusions and inversions of distinct pairs.

## 1. Introduction

The complex geological history and geomorphology of Madagascar have resulted in an exceptional environmental variability that is reflected by its highly diversified biomes and zoocoenoses [1]. In particular, the herpetofauna of Madagascar comprises an extraordinary diversity at different taxonomic levels and a high rate of endemic and microendemic species [2]. Overall, the squamate reptiles of the region include twelve families of snakes (Boidae, Elapidae, Psammophiidae, Pseudoxyrhophiidae, Typhlopidae, and Xenotyphlopidae) and lizards (Agamidae, Chamaeleonidae, Gekkonidae, Gerrhosauridae, Opluridae and Scincidae) with more than 460 described species and several genetic evolutionary lineages that are still awaiting formal description [3]. Among the reptile families from Madagascar, the extant representatives of the family Gekkonidae represent a significant fraction of the total squamate diversity of the island, including more than 140 described species that are subdivided into eleven genera (*Blaesodactylus*, *Ebenavia*, *Geckolepis*, *Gehyra*, *Hemidactylus*, *Lygodactylus*, *Matoatoa*, *Paroedura*, *Paragehyra*, *Phelsuma*, and *Uroplatus*) [3]. With the exception of *Lygodactylus* (which includes representatives from Southern Africa and South America), *Gehyra* (which is mostly distributed between Southeast Asia and Oceania) and *Hemidactylus* (which is one of the most widespread reptile genus worldwide), all other eight gecko genera are endemic to Madagascar and its surrounding islands [4]. Among them, the genus *Paroedura* includes 23 described endemic species of ground geckos from Madagascar and two species from the Comoros [3]. The complex taxonomy of the genus has been updated several times in the last few years with the resurrection and the new description of several species [5,6,7,8].

Recent phylogenetic analyses subdivided the genus *Paroedura* into several main molecular clades or species groups [9,10,11]. In particular, the *Paroedura bastardi* group includes: *P. bastardi*, several molecular clades ascribed to *P. guibeae*, *P. tanjanka*, an undescribed clade named *P*. aff. *tanjanka*, *P. neglecta*, *P. ibityensis*, *P. manongavato* and *P. rennerae* (see [7,8]). In turn, the *P. picta* group includes: *P. picta*, *P. masobe*, *P. androyensis* and *P. maingoka* [5].

In the first chromosome analyses on the genus, Main et al. [12] and Aprea et al. [13] described the karyotypes of 2 and 11 species, respectively, including several taxa of the *P. bastardi* and *P. picta* species groups. These studies show that the genus is characterized by a karyotype composed of 2n = 31–38 mostly acrocentric chromosomes, with NORs always localized on microchromosome pairs and the occurrence of a putative multiple sex chromosome system with female heterogamety (Z_1_Z_1_Z_2_Z_2_/Z_1_Z_2_W) in *P. gracilis*. Further chromosome studies on the genus have evidenced the presence of simple sex chromosome systems with female heterogamety (ZW) in several species (*P. masobe*, *P. karstophila*, *P. oviceps*, *P. stumpffi* and *P. lohatsara*), while a putative multiple sex chromosome system was found in *P. gracilis* (Z_1_Z_1_Z_2_Z_2_/Z_1_Z_2_W_1_W_2_) [14]. The occurrence of this unusual sex chromosome system in *P. gracilis* was hypothesized considering the presence of two female-specific heterochromatic elements (W_1_ and W_2_) and an even chromosome complement (2n = 38) in both males and females [14]. Interestingly, while the simple sex chromosome system of the first five species was considered to be homologous, the absence of heteromorphic sex chromosomes in some species, as well as the derived multiple sex chromosome system in *P. gracilis*, led to the hypothesis of their possible secondary loss in some taxa [14,15].

The emerging karyological scenario identifies the genus *Paroedura* as an excellent study model in evolutionary cytogenetics. In fact, the species so far karyotyped show a significant variability both in terms of chromosome number and morphology, as well as in the diversification of sex chromosomes. However, their taxonomy has been revised since the cytogenetic studies cited above, resulting in a confusing picture.

In this study, we performed a comparative cytogenetic analysis on several representatives of the *P. bastardi* and the *P. picta* species groups, reworking some samples previously studied by Aprea et al. [13], providing new karyological data of some taxa and linking the molecular species delimitation recently performed by Miralles et al. [7] and Piccoli et al. [8] to the available karyotype data.

We describe the chromosome formula and the occurrence of putative heterogametic sex chromosome pairs in additional species of the *P. bastardi* and *P. picta* species groups, including an undescribed molecular clade from Eastern Madagascar. Our results highlight that the observed genetic and cytogenetic diversity of *Paroedura* is still underestimated, and that this genus should be the focus of further molecular and cytogenetic studies. Finally, considering the available cytogenetic data, we offer a hypothesis on the karyotype evolution of the *P. bastardi* and *P. picta* species groups.

## 2. Materials and Methods

### 2.1. Sampling

Nine individuals of the genus *Paroedura* from Madagascar were analyzed in this study. The identification numbers, sampling locality, sex of the samples studied as well as their taxonomic attribution after molecular analysis (see below) are listed in Table 1. The samples were collected during fieldwork conducted between 2002 and 2004 by various collaborators and no animal was sampled during the realization of this study. After capture, the individuals were injected with a colchicine solution of 0.5 mg/mL (0.1 mL/10 g of body weight). Tissue samples were then incubated for 30 min in hypotonic solution (KCl 0.075 M + sodium citrate 0.5%, 1:1), and fixed and preserved in Carnoy’s solution (methanol/acetic acid, 3:1). The fixed biological material was stored at 4 °C and transferred to the laboratory, where it was processed as described below. The experimental procedures described below were conceived to provide new molecular and chromosome data on the genus *Paroedura*, also by filling some informational gaps left by Aprea et al. [13] (e.g., missing DNA sequences and/or karyotypes). The taxonomic attribution of the study samples provided in Table 1 was determined by means of a preliminary molecular analysis using a trait of the 16S rDNA (16S) (samples GA324, GA325, GA505, GA388, FGMV1523, FGMV1524) (see below) or using homologous traits previously provided by Aprea et al. [13] (for samples GA506, GA389 and FGMV1522).

### 2.2. Molecular Analysis

We performed a preliminary molecular analysis in order to assess the taxonomic identity of the samples analyzed and to link the available DNA sequences to the newly described karyotypes. In particular, the molecular procedures described as follows were realized on the samples GA 388 and GA 505 (which were not analyzed with molecular methods in Aprea et al. [13]) and on the samples FGMV 1523, FGMV 1524, GA 324 GA 325 (which were not analyzed with either molecular or cytogenetic methods in Aprea et al. [13]). Genomic DNA was extracted from cell suspensions and tissue samples using the standard phenol–chloroform reported in Sambrook et al. [16].

A fragment of the mitochondrial 16S rRNA gene of about 571 nucleotidic positions was amplified using the primer pairs 16Sa 5′–AAACTGGGATTAGATACCCCACTAT–3′ (forward) and 16Sb 5′–GAGGGTGACGGGCGGTGTGT–3′ (reverse) [17]. This molecular marker was selected with consideration of its wide application on the genus *Paroedura*, the number of DNA sequences deposited on GenBank, and the DNA sequences provided by Aprea et al. [13], Cocca et al. [18], Miralles et al. [7] and Piccoli et al. [8].

PCR was performed in 25 μL of reaction volume and using the following cycle conditions: an initial denaturation step at 94 °C for 5 min, followed by 36 cycles of denaturation at 94 °C for 30 s, annealing at 55 °C for 30 s, extension at 72 °C for 45 s and a final elongation step at 72 °C for 7 min. The resulting amplicons were then sequenced using the BigDye Terminator 3.1 kit (ABI) and an ABI 377 automated sequencer (Applied Biosystems, Foster City, CA, USA). The chromatograms obtained were visually checked and edited with Chromas Lite 2.6.6 and BioEdit 7.7.1 [19]. To perform a molecular taxonomic attribution, all the newly generated DNA sequences were blasted on GenBank and compared with available homologous sequences, previously used in taxonomic and phylogenetic studies on the genus *Paroedura* [5,7,8,9,11,13]. The sequences with an identity score ≥ 98% (uncorrected *p*–distance) were considered to be conspecific.

### 2.3. Cytogenetic Analysis

Chromosomes in metaphase were obtained using the air-drying technique as reported by Mezzasalma et al. [20]. Metaphase plates were stained with standard methods (10 min in a solution of 5% Giemsa at pH 7), silver (Ag–NOR) staining [21], C-banding as described by Sumner [22] and sequential C-banding + CMA_3_ and + DAPI [23]. Metaphase plates were scored and recorded by means of an optical and an epifluorescence microscope (Axioscope Zeiss, Oberkochen, Germany) equipped with an image analysis system. The reconstruction of karyotypes and the calculation of the chromosome relative length (RL = chromosome length/total length of the karyotype) and centromeric index of each chromosome (CI = short arm length/total length of the chromosome) were then performed after the acquisition of at least 15 metaphase plates per individual studied, and chromosomes were classified following the categories proposed by Levan et al. [24], as metacentric (m), submetacentric (sm), subtelocentric (st) or acrocentric (a).

## 3. Results

### 3.1. Molecular Analysis

The selected fragments of the 16S were successfully amplified in all the samples used in the molecular analysis (GA 324, GA 325, GA 388, GA 505, FGMV 1523, FGMV 1524). All the newly generated sequences were deposited in GenBank (accession numbers: PP836632–37). The sample GA 388 showed an identity score > 99% with the sample GA 389 (Accession number: GU129006, see [13]), which was taxonomically identified as *P. ibityensis* in Miralles et al. [7]. The sample GA 505 showed an identity score of 100% with the sample GA 506 (accession number: GU128989, see [13]) and >98% with samples ZSM849/2010 (ZCMV 12740) and GA 374 (accession numbers: MW318987 and GU129005, see [7,13]), ascribed to *P. rennerae* in Miralles et al. [7]. The samples FGMV 1523 and FGMV 1524 had the same haplotype, showing an identity score of 100% with the sample ZMA 19603 (accession number: GU128992, see [13]) and of 90.1% with samples MRSN: R2528 and MRSN: R2529 (accession numbers: MH063302 and MH063303, see [18]). It should be noted that both samples FGMV 1523 and FGMV 1524 were used in the taxonomic and molecular analysis by Miralles et al. [7], where they were identified as *P. guibeae*. The samples GA 324 and GA 325 had the same haplotype, which showed an identity score of about 94–95% with the specimens FGMV 1522, FGMV 2236 and ACP2541 (accession numbers: GU128988, GU128991), ascribed to *P. picta* in Aprea et al. [13] and Cocca et al. [18], and with the homologous 16S trait of the complete mitochondrial genomes sequenced in Hara et al. [25] (accession number: AP019518) and Starostová and Musilová [26] (accession number: NC_028326).

### 3.2. Cytogenetic Analysis

**Figure 1:**
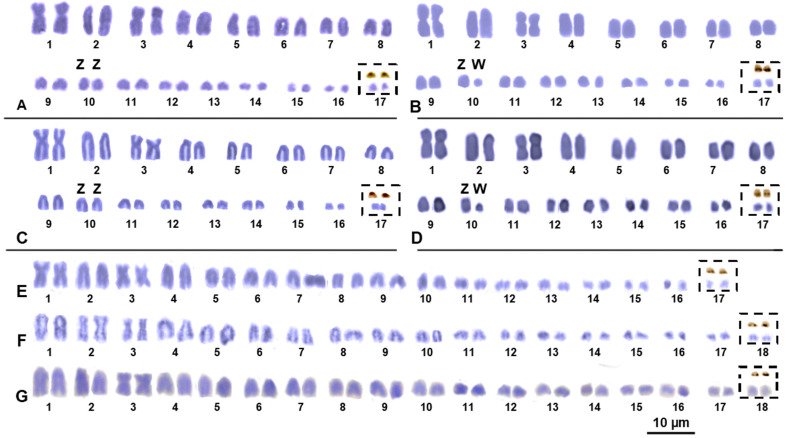
Giemsa-stained karyotypes of male (**A**) and female individuals (**B**) of *P. ibityensis*, of male (**C**) and female individuals (**D**) of *P. rennerae*, and female individuals of *P.* cf. *guibeae* (**E**), *P*. cf. *picta* (**F**) and *P. picta* (**G**). The boxes show the NOR bearing pair, stained with Giemsa (down) and Ag–NOR staining (up). The scale bar applies to all images.

The reconstruction of karyotypes with standard staining (5% Giemsa solution at pH 7) was successfully performed on all the samples studied. Our results highlight that the karyotype of all the studied individuals of the *P. bastardi* species group (*P. ibytensis*, *P. rennerae* and *P.* cf. *guibei*) (see Table 1) have a similar chromosome formula of 2n = 34 chromosomes, which gradually decrease in size without showing any clear distinction between macro- and microchromosome pairs (Figure 1).

These species also show a similar chromosome morphology with two metacentric chromosomes (here represented as chromosome pairs 1 and 3), while all the remaining pairs are acrocentric (Figure 1). The female specimens of *P. ibytiensis*, and *P. rennerae* showed a dimensionally heteromorphic chromosome pair (here described as chromosome pair 10), which was also more evident after C-banding (see below). No heteromorphic chromosome pair was detected in the male samples of the same species or in *P. guibeae* (Figure 1). The two specimens of *P. picta* and *P.* cf. *picta* both show a chromosome complement of 2n = 36, mostly composed of acrocentric chromosome pairs that are gradually decreasing in size (Figure 1). However, in *P. picta*, one chromosome pair is metacentric (pair 3), while in *P.* cf. *picta*, two pairs are metacentric (pairs 2 and 3) (Figure 1). No heteromorphic chromosome pair was observed in the studied female samples of *P. picta* and *P.* cf. *picta* (Figure 1).

After Ag–NOR staining, all the studied species of *Paroedura* showed paired NOR loci on a small chromosome pair (here depicted as the last chromosome pair) (Figure 1).

**Figure 2:**
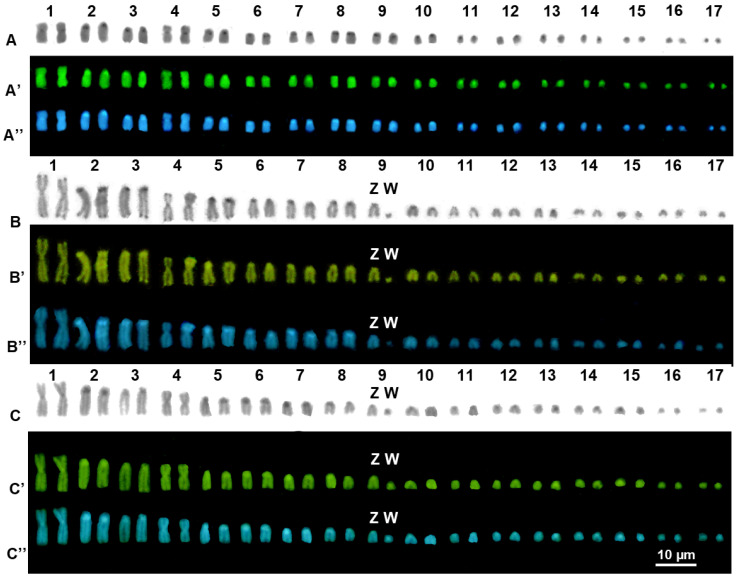
C-banded karyotypes of female individuals of *P.* cf. *guibeae* (**A**), *P. ibityensis* (**B**) and *P. rennerae* (**C**), with C-banding + Giemsa (**A**–**C**), + CMA3 (**A′**–**C′**) and + DAPI (**A″**–**C″**).

**Figure 3:**
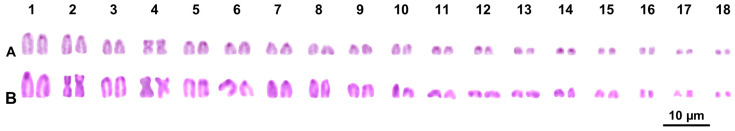
Karyotypes of female individuals of *P. picta* (**A**) and *P.* cf. *picta* (**B**) with C-banding + Giemsa.

C-banding and sequential C-banding evidenced a generally low content of heterochromatin in all the studied species (Figure 2 and Figure 3). 

Distinct heterochromatic blocks were mostly present on telomeric, centromeric and pericentromeric regions of most chromosome pairs, which, in *P. ibityensis*, *P. rennerae* and *P.* cf. *guibeae*, were evident with bot C-banding + Giemsa and C-banding + fluorochromes (Figure 2). In *P. picta* and *P.* cf. *picta,* heterochromatic blocks were more evident with C-banding + Giemsa (Figure 3) than with C-banding + fluorochromes. Furthermore, in *P. ibytensis* and *P. rennerae*, the small element of the heteromorphic pair 10 proved to be largely heterochromatic, possibly corresponding to the heterogametic chromosome of a sex chromosome pair with female heterogamety (ZW). Interestingly, the W element of *P. ibytensis* appeared relatively smaller than the W chromosome of *P. rennerae* (Figure 1 and Figure 2). No heteromophic chromosome was found in *P.* cf. *guibeae* (Figure 2) or in *P. picta* and *P.* cf. *picta* (Figure 3).

## 4. Discussion

### 4.1. Molecular Analysis

Our molecular results help us to assess the taxonomic status of the specimens of the *P. bastardi* and *P. picta* species groups with available cytogenetic data. In particular, we are confident about the taxonomic attribution of *P. ibityensis* (for samples GA 388 and GA 389) and *P. rennerae* (for samples GA 505 and GA 506), which showed a sequence identity score > 98% with different specimens that have been recently used in taxonomic and phylogenetic studies on the genus (see [7,8]). In turn, our results on *P.* cf. *guibeae* show (also according to the phylogenetic relationships provided in [7]) the existence of distinct molecular clades ascribed to *P. guibeae*, which probably deserve a focused taxonomic assessment and might represent a species complex. In fact, these different molecular clades (respectively, from Isalo, Tranoroa and Toliara) form three distinct branches in the phylogenetic tree provided by Miralles et al. [7], showing significant haplotype diversity. The specimens analyzed in this study (FGMV 1523 and FGMV 1524), which were also incorporated in the phylogenetic analysis in [7]), belong to the Toliara clade. However, because no molecular data are available for specimens from the type locality of the species (Betroka), it is uncertain which molecular clade corresponds to *P. guibeae s.s.*, and we here prefer to refer to the samples analyzed in this study as *P.* cf. *guibeae*. 

We also highlight that the available karyotypes, previously ascribed to *P. bastardi* in Aprea et al. [13], correspond to *P. ibityensis* (GA 388 and GA 389), *P. rennerae* (GA 374, GA 505, GA 506), and *P.* cf. *guibeae* (MRSN R2415, ZMA 19603). Similarly, the deposited sequences linked to the karyotype described as belonging to *P. bastardi* (AN: KJ917172) and to *P. ibityensis* (AN: KJ917173) in Koubová et al. [14] also correspond to *P.* cf. *guibeae.* Therefore, to the best of our knowledge, no karyotype is currently available for *P. bastardi*.

Interestingly, we also found a significant haplotype diversification between available sequences of *P. picta* and the samples GA 324 and GA 325, which are here referred to as *P.* cf. *picta*, also considering the karyotype differences observed (see Results). We chose to refer to sample FMGV 1522 as *P. picta* and samples GA 324 and GA 325 as *P.* cf. *picta,* considering that the locality reported for the former is geographically closer to the type locality of the species (Madagascar, St. Augustins Bay) [27].

### 4.2. Cytogenetic Analysis

Chromosome data may provide valuable taxonomic and evolutionary information on the taxa studied. In fact, different chromosome characters (e.g., number, morphology, localization of particular chromosome markers and absence/presence of differentiated sex chromosomes) can represent ancestral or apomorphic states, which can be useful to understanding evolutionary dynamics (see e.g., [20,28,29,30]).

**Figure 4:**
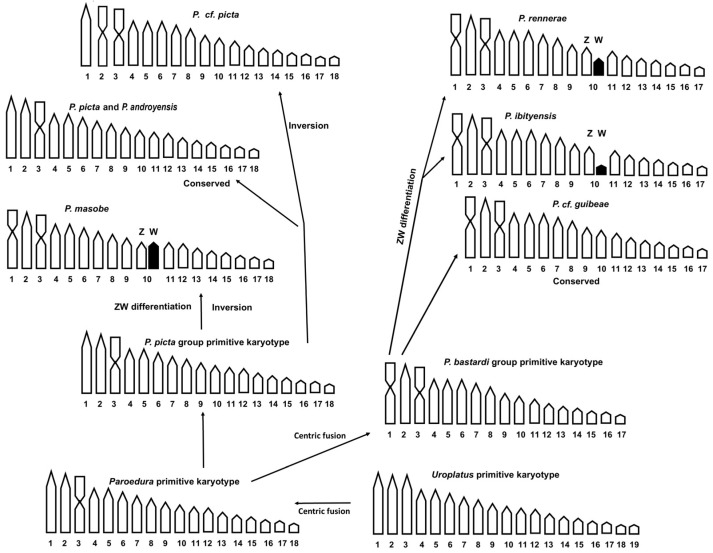
Hypothesized pathways of chromosome diversification in the *P. bastardi* and *P. picta* species groups.

Overall, our results highlight that the karyotype variability in the *P. bastardi* and *P. picta* species groups involves chromosome number (2n = 34–36), chromosome morphology (with a different complement of metacentric and telocentric elements) and the independent diversification of ZZ/ZW heterogametic sex chromosome pairs (Figure 4). The three available karyotypes of the *P. bastardi* group (*P. ibityensis*, *P. rennerae* and *P.* cf. *guibeae*) all show a similar karyotype with 2n = 34 characterized by two metacentric pairs (1 and 3), which mostly differ by the presence/absence and diversification stage of a ZW heteromorphic chromosome pair (Figure 4).

In fact, a similar putative ZZ/ZW sex chromosome pair was detected in this study in both *P. ibityensis* and *P. rennerae*, where the W chromosome appears largely heterochromatic and smaller than the Z chromosome. The relatively smaller size of the W of *P. ibityensis* (see Results) can be explained by the progressive degeneration of the heterochromatic sex chromosome, which is commonly observed in several evolutionary lineages (see e.g., [31]). It should be noted that the differentiation of heterogametic sex chromosomes in these species was not previously highlighted in Aprea et al. [13], probably because only standard chromosome staining was performed on representatives of those species. In Koubová et al. [14], sex chromosomes were also not reported for *P. ibityensis*, but as already specified above, the deposited DNA sequence associated to this species corresponds to *P. guibeae*, which was later resurrected in Miralles et al. [7]. Concerning the specimens here identified as *P.* cf. *guibeae*, we also did not find any evidence of a differentiated sex chromosome system, thus confirming previous observations and suggesting that sex chromosomes in this species are probably in an early stage of diversification, and mostly pseudo-autosomal. In the *P. picta* species group, no differentiated sex chromosomes were observed in *P. picta* or *P.* cf. *picta*, but a ZZ/ZW sex chromosome system was reported by Koubová et al. [14] in *P. masobe* (Figure 4). As previously pointed out in Koubová et al. [14] and Rovatsos et al. [15], different possible explanations can be hypothesized in order to explain the absence/presence of heteromorphic sex chromosome pairs and the occurrence of different stages of diversification in the genus *Paroedura*. The first hypothesis implies the reversal of differentiated sex chromosomes back to the undifferentiated state (with a possible sex chromosome turnover), while the second accounts for different diversification rates in distinct clades (see [15]).

Neither hypothesis can be discarded on the basis of the available karyological and molecular data, but we consider the occurrence of homologous sex determination systems at different stages of diversification as the most likely and parsimonious scenario in *Paroedura*. In fact, sex chromosome pairs usually begin their evolution as highly homologous, pseudo-autosomal elements, and their diversification rate (as in other chromosome or nucleotide changes) can be dependent on the occurrence of particular events (e.g., macromutation followed by heterochromatinization and heterochromatin degradation), which are stochastic in nature [31,32,33]. As a result, phylogenetically closely related species or even populations may present sex chromosomes that are variable in morphology, heterochromatin content and distribution (see e.g., [34,35,36,37]).

It should also be stressed that the general karyotype structure observed in *Paroedura* closely resembles that reported for the phylogenetically related geckos of the genera *Uroplatus*, *Matoatoa*, *Lygodactylus*, *Ebenavia*, *Christinus* and *Phelsuma*. In fact, all these gecko genera show a karyotype composed of 2n = 34–42 mostly acrocentric elements, with a tendency towards the formation of biarmed elements via tandem fusions and chromosome inversions (see e.g., [37,38,39,40]). 

Considering several characters that are regarded as ancestral karyological features in squamates (e.g., relatively higher total number of chromosomes, ratio of biarmed and acrocentric elements and loci on NORs on the smallest chromosome pairs (see e.g., [39,41,42])), a karyotype of 2n = 38 with all acrocentric elements was recently hypothesized as the ancestral condition in *Uroplatus* [37] (Figure 4). Starting from a similar karyotype, one tandem fusion would have originated a karyotype of 2n = 36 with one metacentric pair, which is highly represented in *Paroedura* (see also [13,14]) and can be likely considered as the ancestral condition in the genus (Figure 4). Among the species here studied, this hypothesized ancestral condition would have been conserved in *P. picta* and *P. androyensis*, while the different morphologies of the first and second chromosome pair in *P. masobe* and *P.* cf. *picta*, respectively, likely involved two distinct chromosome inversions (Figure 4). In turn, the karyotype of 2n = 34, which appears to be conserved in the *P. bastardi* group, probably originated from the *Paroedura* ancestral karyotype by means of a tandem fusion (Figure 4).

This evolutionary hypothesis also shows that a comparable pathway of diversification can be observed in the karyotype of different evolutionary lineages of the family Gekkonidae. Indeed, a decrease in the total chromosome number and the progressive formation of biarmed elements via chromosome fusions and inversions is overall one of the most frequently observed karyotype dynamics in squamates (see [42,43,44,45,46,47]). In the genus *Paroedura*, some chromosome rearrangements should be considered non-homologous, involving different chromosome pairs and potentially driving processes of speciation and lineage diversification.

## 5. Conclusions

The intrageneric chromosome variability of the Malagasy ground geckos of the genus *Paroedura* involves chromosome number, morphology, and the diversification of sex chromosome systems with female heterogamety (ZZ/ZW). In the *P. bastardi* species group, chromosomal diversification is apparently mainly linked to the independent differentiation of heterogametic sex chromosome systems, while in two taxa of the *P. picta* group a different morphology of the second chromosome pair is likely due to an inversion. The general karyotype structure of the genus shares many different features in common with phylogenetically related gecko genera, including the prevalence of acrocentric elements and the progressive formation of biarmed elements by means of chromosome fusions and fissions, which can often be described as non-homologous. Although the genus *Paroedura* has been the focus of several molecular and cytogenetic studies, our results underline that its taxonomic, genetic and chromosomal diversity should still be considered underestimated. 

## Figures and Tables

**Table 1:** Taxonomic attribution, origin and sex of the studied samples of the genus *Paroedura*.

Species	Specimen	Locality	Sex
*P. ibytiensis*	GA 388	Mount Ibity	female
*P. ibytiensis*	GA 389	Mount Ibity	male
*P. rennerae*	GA 505	Mandrivazo	female
*P. rennerae*	GA 506	Mandrivazo	male
*P.* cf. *guibeae*	FGMV1523	Toliara	male
*P.* cf. *guibeae*	FGMV1524	Toliara	female
*P.* cf. *picta*	GA 324	Marofandilia	female
*P.* cf. *picta*	GA 325	Marofandilia	female
*P. picta*	FGMV1522	Toliara region	female

## Data Availability

The newly generated DNA sequences are available on GenBank (accession numbers: PP836632–37).
